# Rare genetic variants in the gene encoding histone lysine demethylase 4C (*KDM4C*) and their contributions to susceptibility to schizophrenia and autism spectrum disorder

**DOI:** 10.1038/s41398-020-01107-7

**Published:** 2020-12-05

**Authors:** Hidekazu Kato, Itaru Kushima, Daisuke Mori, Akira Yoshimi, Branko Aleksic, Yoshihiro Nawa, Miho Toyama, Sho Furuta, Yanjie Yu, Kanako Ishizuka, Hiroki Kimura, Yuko Arioka, Keita Tsujimura, Mako Morikawa, Takashi Okada, Toshiya Inada, Masahiro Nakatochi, Keiko Shinjo, Yutaka Kondo, Kozo Kaibuchi, Yasuko Funabiki, Ryo Kimura, Toshimitsu Suzuki, Kazuhiro Yamakawa, Masashi Ikeda, Nakao Iwata, Tsutomu Takahashi, Michio Suzuki, Yuko Okahisa, Manabu Takaki, Jun Egawa, Toshiyuki Someya, Norio Ozaki

**Affiliations:** 1 Department of Psychiatry, Nagoya University Graduate School of Medicine, Nagoya, Japan; 2 Medical Genomics Center, Nagoya University Hospital, Nagoya, Japan; 3 Brain and Mind Research Center, Nagoya University, Nagoya, Japan; 4 Division of Clinical Sciences and Neuropsychopharmacology, Faculty and Graduate School of Pharmacy, Meijo University, Nagoya, Japan; 5 Center for Advanced Medicine and Clinical Research, Nagoya University Hospital, Nagoya, Japan; 6 Innovative Research Unit for Developmental Disorders, Institute of Advanced Research, Nagoya University, Nagoya, Japan; 7 Public Health Informatics Unit, Department of Integrated Health Sciences, Nagoya University Graduate School of Medicine, Nagoya, Japan; 8 Division of Cancer Biology, Nagoya University Graduate School of Medicine, Nagoya, Japan; 9 Department of Cell Pharmacology, Nagoya University Graduate School of Medicine, Nagoya, Japan; 10 Department of Cognitive and Behavioral Science, Graduate School of Human and Environmental Studies, Kyoto University, Kyoto, Japan; 11 Department of Anatomy and Developmental Biology, Graduate School of Medicine, Kyoto University, Kyoto, Japan; 12 Department of Neurodevelopmental Disorder Genetics, Institute of Brain Science, Nagoya City University Graduate School of Medical Sciences, Nagoya, Japan; 13 Laboratory for Neurogenetics, RIKEN Center for Brain Science, Saitama, Japan; 14 Department of Psychiatry, Fujita Health University School of Medicine, Toyoake, Japan; 15 Department of Neuropsychiatry, University of Toyama Graduate School of Medicine and Pharmaceutical Sciences, Toyama, Japan; 16 Research Center for Idling Brain Science, University of Toyama, Toyama, Japan; 17 Department of Neuropsychiatry, Okayama University Graduate School of Medicine, Dentistry and Pharmaceutical Sciences, Okayama, Japan; 18 Department of Psychiatry, Niigata University Graduate School of Medical and Dental Sciences, Niigata, Japan

**Keywords:** Medical genetics, Epigenetics in the nervous system

## Abstract

Dysregulation of epigenetic processes involving histone methylation induces neurodevelopmental impairments and has been implicated in schizophrenia (SCZ) and autism spectrum disorder (ASD). Variants in the gene encoding lysine demethylase 4C (*KDM4C*) have been suggested to confer a risk for such disorders. However, rare genetic variants in *KDM4C* have not been fully evaluated, and the functional impact of the variants has not been studied using patient-derived cells. In this study, we conducted copy number variant (CNV) analysis in a Japanese sample set (2605 SCZ and 1141 ASD cases, and 2310 controls). We found evidence for significant associations between CNVs in *KDM4C* and SCZ (*p* = 0.003) and ASD (*p* = 0.04). We also observed a significant association between deletions in *KDM4C* and SCZ (corrected *p* = 0.04). Next, to explore the contribution of single nucleotide variants in *KDM4C*, we sequenced the coding exons in a second sample set (370 SCZ and 192 ASD cases) and detected 18 rare missense variants, including p.D160N within the JmjC domain of KDM4C. We, then, performed association analysis for p.D160N in a third sample set (1751 SCZ and 377 ASD cases, and 2276 controls), but did not find a statistical association with these disorders. Immunoblotting analysis using lymphoblastoid cell lines from a case with *KDM4C* deletion revealed reduced KDM4C protein expression and altered histone methylation patterns. In conclusion, this study strengthens the evidence for associations between *KDM4C* CNVs and these two disorders and for their potential functional effect on histone methylation patterns.

## Introduction

Schizophrenia (SCZ) and autism spectrum disorder (ASD) are considered clinically different psychiatric disorders in the Diagnostic and Statistical Manual of Mental Disorders, Fifth Edition (DSM-5)^1^. Nevertheless, recent studies indicate an overlap between the two disorders in genetic risk factors, biological pathways, and phenotypic features including deficits in social-cognitive functioning^2,3^. Strong evidence indicates that genetic factors contribute substantially to the etiology of SCZ and ASD, with heritability based on twin study estimates as high as 80% for SCZ^4^ and 90% for ASD^5^. Recent genome-wide studies of SCZ and ASD revealed that rare copy number variations (CNVs) and single nucleotide variations (SNVs) may have large effect sizes and that elucidating the pathophysiology of these disorders may be possible^3,6–9^.

Recent CNV studies and exome studies provide increasing evidence that numerous genes are involved in SCZ and ASD, including genes associated with epigenetic processes involving post-translational histone lysine methylation^3,10–13^. Multiple genes encode histone demethylases and histone methyltransferases, which regulate histone 3 lysine 9 (H3K9) methylation and H3K36 methylation (e.g., *EHMT1*, *KDM3A*, *KDM4B*, *NSD1*, and *SETDB1*). Di-methylated histone 3 lysine 9 (H3K9me2) levels are higher in both lymphocytes and postmortem brain obtained from SCZ patients compared to nonpsychiatric controls^14,15^. Studies using histone demethylase and histone methyltransferase mutant mice revealed that methylation patterns at H3K9 and H3K36 regulate the expression of genes related to neural functions in brain^16,17^. Furthermore, these studies showed deficits in neural maturation^16,18^, synaptic dysfunction^19^, and behavioral characteristics linked to SCZ and ASD^20–22^ (e.g., diminished social behavior and learning and memory deficits). These findings suggest that these enzymes that target H3K9 and H3K36 play an important role in neural development and neural function. Lysine demethylase 4C, encoded by *KDM4C*, demethylates H3K9me2/me3 and H3K36me2/me3^23,24^. KDM4C is expressed from fetal to adult stages^25^ and regulates differentiation of neural stem cells^26^, suggesting the significance of KDM4C for early neural development. *Kdm4c* hypomorphic mutant mice exhibit abnormal behaviors such as hyperactivity, persistence, and learning and memory deficits^27^. Some of the phenotypic features of these mice resemble the characteristics of developmental disorders including ASD. Furthermore, several SCZ and ASD cases with *KDM4C* CNVs have been reported^28,29^, and we recently reported that rare CNVs of *KDM4C* are associated with SCZ and ASD in a Japanese population^3^.

Considering that KDM4C plays an important role in neurodevelopment and has been linked to SCZ and ASD, we hypothesized that rare CNVs and SNVs in *KDM4C* may confer susceptibility to these disorders. However, *KDM4C* variants have not been fully evaluated by CNV analysis or deep sequencing of samples from ASD and SCZ. First, in a previous genome-wide CNV study^3^, to reduce false positives, we excluded some putative CNVs according to stringent criteria for CNV calling and quality control (QC). Thus, the statistical power was not sufficient to evaluate SCZ and ASD separately. Second, the breakpoints of the CNVs were not evaluated in the previous study. Third, the phenotypic features of cases with *KDM4C* CNVs have not been evaluated. Fourth, no sequencing studies have focused on *KDM4C* variants by deep sequencing of samples from SCZ and ASD. Finally, the functional impact of *KDM4C* CNVs has not been studied using cells derived from clinical cases with such variants. Therefore, in the present study, we analyzed *KDM4C* CNVs with moderate criteria for CNV calling and QC in an expanded sample set. Then, we evaluated the genetic association between the CNVs and SCZ or ASD. We also determined the breakpoints of the CNVs and assessed clinical phenotypes of patients with such CNVs. Furthermore, to discover novel SNVs in *KDM4C* associated with such disorders, we sequenced the *KDM4C* coding exons in patients with ASD and SCZ, and then performed an association analysis. Finally, we evaluated the change in gene expression of KDM4C, and histone methylation patterns using lymphoblastoid cell lines (LCLs) established from SCZ patients with *KDM4C* CNVs.

## Methods

### Subjects

All subjects were living in Japan and self-identified as Japanese. Cases were diagnosed according to DSM-5 criteria for SCZ or ASD. In the CNV analysis, we used the first sample set, which comprised 2810 SCZ cases (mean age = 46.0 ± 16.3 years; males = 55.1%), 1182 ASD cases (mean age = 20.7 ± 10.5 years; males = 77.2%), and 2428 healthy controls (mean age = 38.8 ± 14.9%; males = 49.6%). In the screening of rare variants of *KDM4C*, we used the second sample set, which comprised 370 SCZ cases (mean age = 49.7 ± 14.7 years; males = 52.9%) and 192 ASD cases (mean age = 16.3 ± 8.4 years; males = 77.6%), was sequenced for screening of rare variants of *KDM4C*. In association analysis of a missense variant, we analyzed the third sample set, which comprised 1751 SCZ cases (mean age = 47.8 ± 15.3 years; males = 53.7%), 377 ASD cases (mean age = 19.1 ± 10.2 years; males = 77.5%) and 2276 controls (mean age = 44.8 ± 15.1 years; males = 50.9%). Control subjects were selected from the general population and had no history of mental disorders based on questionnaire responses from the subjects themselves during the sample inclusion step. This study was approved by the ethics committee of the Nagoya University Graduate School of Medicine and other participating institutes, and written informed consent was obtained from all participants. In addition, the patient’s capacity to consent was confirmed by a family member when needed.

### Array comparative genomic hybridization (aCGH) and CNV analysis

Genomic DNA was extracted from blood and/or saliva samples. aCGH was performed using two types of arrays: the NimbleGen CGH Array 720K (Roche NimbleGen, Madison, WI, USA) and the Agilent 400K CGH Array (Agilent Technologies, Santa Clara, CA, USA). For both, CNV calls were made with Nexus Copy Number software v9.0 (BioDiscovery) using the Fast Adaptive States Segmentation Technique 2 algorithm. To obtain CNV calls, log2 ratio thresholds for the loss and gain were set at −0.4 and 0.3, respectively. The threshold for a significant *p* value was set at 1 × 10^−3^ and at least three contiguous probes were required for CNV calls. Using these settings, the CNV detection resolution of the two types of arrays was similar. A noise-reduction algorithm for the aCGH data was used as a systematic correction of artifacts caused by GC content or fragment length. We calculated the QC scores for each sample based on the statistical variance of the probe-to-probe log ratios and removed samples with QC > 0.15. We also removed samples with excess numbers of CNVs. Then we excluded CNVs <10 kb, those with >50% overlap with segmental duplications, and those with Call *P* Value > 1 × 10^−6^. After filtering out common CNVs (≥1% of the total sample), exonic CNVs were identified in *KDM4C*.

All genomic locations are given in NCBI build 36/UCSC hg18 coordinates because the arrays used were designed based on hg18. We performed all analyses based on the gene annotation from GENCODE version 31 ^30^, the genomic coordinates of which were converted to hg18 using the UCSC LiftOver tool (http://genome.ucsc.edu/cgi-bin/hgLiftOver).

Copy number changes were confirmed with quantitative RT-PCR (qPCR) analysis using QIAGEN qBiomarker Copy Number PCR assays (Qiagen Ltd., Hilden, Germany). qBiomarker Copy Number PCR Assays were designed for exons 1 and 4 of *KDM4C* (NM_001146696.2) (Supplementary Table S1). A qBiomarker Multicopy Reference Copy Number PCR Assay (MRef) (Qiagen Ltd.) was included in each assay. qPCR was performed with a Quant Studio 5 Real-Time PCR System (Applied Biosystems, Foster City, CA, USA) using KAPA SYBR FAST qPCR Master Mix (Kapa Biosystems, Wilmington, MA, USA). Data were analyzed with free data analysis software for the qBiomarker Copy Number PCR Assay available at https://www.qiagen.com/hk/shop/genes-and-pathways/data-analysis-center-overview-page/.

### Breakpoint analysis

We performed PCR to search for the breakpoints of the CNVs. We designed primers that aligned with the region of the breakpoints (Supplementary Table S2). The PCR products were sequenced by using the ABI 3130XL Genetic Analyzer (Applied Biosystems) according to the standard protocols.

### Detection of SNVs and association analysis

We extracted genomic DNA from whole peripheral blood and/or saliva according to standard methods. To cover coding regions of *KDM4C* (human reference sequence NCBI (build 37)), we designed custom amplification primers with FastPCR (PrimerDigital Ltd., Helsinki, Finland) and NCBI Primer-BLAST. The Ion Library Equalizer Kit Adapters and Ion AmpliSeq Library Kits 2.0 (Thermo Fisher Scientific, Waltham, MA, USA) were used for amplification and equalization. Then Ion Xpress Barcode was used to collect the amplified sequence. We used Ion Torrent PGM™ (Thermo Fisher Scientific) to sequence the products using next-generation sequencing technology. Then we performed an analysis for the resulting data using Ingenuity Variant Analysis (Qiagen Ltd.). We prioritized SNVs for follow-up association analysis as follows. We selected rare (minor allele frequency ≤1%) nonsynonymous missense variants. Each rare variant was reconfirmed with Sanger sequencing using the ABI 3130XL Genetic Analyzer (Applied Biosystems). Primer sequences for validating each variant are available in Supplementary Table S3. After prioritization, all variants were evaluated in silico for possible structural and functional consequences using the following tools: (1) deleterious effects by amino acid substitution was predicted by PolyPhen-2 (http://genetics.bwh.harvard.edu/pph2/)^21^ and Sorting Tolerant from Intolerant (SIFT)^22^; and (2) evolutionary conservation was assessed with the HomoloGene database (http://www.ncbi.nlm.nih.gov/homologene/). To investigate the association of discovered rare variants with susceptibility to SCZ and ASD, we prioritized novel variants not documented in the NCBI dbSNP database (Build 137) (http://www.ncbi.nlm.nih.gov/SNP/), Tohoku Medical Megabank Organization (ToMMo) (https://www.megabank.tohoku.ac.jp/), the Human Genetic Variation Database (HGVD) of Japanese genetic variation consortium (http://www.genome.med.kyoto-u.ac.jp/SnpDB), or the Exome Aggregation Consortium (ExAC) (http://exac.broad institute.org). Localization of the protein domain was based on the Pfam protein families database (https://pfam.xfam.org/).

In association analysis of SNVs, genotyping was performed using TaqMan assays with custom probes (Supplementary Table S4), the Quant Studio 5 Real-Time PCR System (Applied Biosystems), and QuantStudio Design and Analysis Software v1.4 (Applied Biosystems) according to the standard protocols.

### mRNA expression analysis using LCLs

LCLs (human lymphocytes transformed with Epstein−Barr virus) from the two cases with *KDM4C* CNVs, 14 SCZ cases without *KDM4C* variants (overlapping with the CNV discovery cohort, mean age ± standard deviation, 40.9 ± 11.9 years; males = 42.9%) and 15 control subjects (mean age ± standard deviation, 45.3 ± 10.7 years; males = 53.3%) were prepared and cultured according to standard protocols. Total RNA was extracted from LCLs using RNAqueous Kit (Ambion, Austin, TX, USA) and treated with DNase to remove contaminating genomic DNA using the TURBO DNA-free Kit (Ambion); RNA was then reverse transcribed to cDNA with the High Capacity RNA-to-cDNA Kit (Applied Biosystems). β-2-Microglobulin (*B2M*) and glucuronidase-β (*GUSB*), two housekeeping genes, were selected as internal control genes. qPCR was performed on the Quant Studio 5 Real-Time PCR System (Applied Biosystems) using KAPA SYBR FAST qPCR Master Mix (Kapa Biosystems). Measurement of the cycle threshold was performed in triplicate. Primer sequences for qPCR are available in Supplementary Table S5. The relative expression was analyzed according to the comparative cycle threshold (Ct) method.

### Immunoblotting analysis

The LCLs were washed with cold PBS and lysed in SDS sample buffer (2% SDS, 10% glycerol, 5% 2-mercaptoethanol, 0.01% bromophenol blue, and 0.1 M Tris HCl, pH 6.8). Following SDS-PAGE, separated proteins were transferred onto polyvinylidene difluoride membranes (Millipore, Billerica, MA, USA). The membranes were blocked with Block Ace (Yukijirushi Corp., Sapporo, Japan) and incubated with anti-KDM4C (rabbit polyclonal, A300-885A, Bethyl Laboratories, Montgomery, TX, USA), anti-H3K4me3 (rabbit polyclonal, ab8580; Abcam, Cambridge, UK), anti-H3K9me2 (mouse monoclonal, ab1220, Abcam), anti-H3K9me3 (rabbit polyclonal, ab8898, Abcam), or anti-H3K36me3 (rabbit polyclonal, ab9050, Abcam). Anti-H3 (rabbit polyclonal, ab1791, Abcam) or anti-GAPDH (rabbit polyclonal, #2118: Cell Signaling Technology, Danvers, MA, USA; mouse monoclonal, M171-3 3H12: MBL, Nagoya, Japan) was used as the loading control. Then the membranes were incubated with Alexa 680- and/or Alexa 800-conjugated secondary antibodies. The specific proteins of interest were imaged and quantitated with Odyssey CLx (LI-COR Biosciences, Lincoln, NE, USA).

### Evaluation of clinical characteristics

The clinical features of patients with *KDM4C* CNVs were obtained retrospectively from medical records. All psychiatric comorbidities were diagnosed by experienced psychiatrists according to DSM-5 criteria.

### Statistics

We calculated the statistical power by using the Genetic Power Calculator (http://zzz.bwh.harvard.edu/gpc/)^31^. One-sided Fisher’s exact test for count data and Bonferroni’s adjustment for multiple comparisons were applied when appropriate to measure significant differences in *KDM4C* CNVs between patients and controls. If no variants were observed in a cell on the 2 × 2 table, odds ratio (OR) was calculated after a 0 cell correction (0.5 was added to all cells) to reduce bias in estimating OR^32^. Differences were considered significant when the *p* value was <0.05. Expression levels in subjects with *KDM4C* CNVs were compared with those in the SCZ group without CNVs or the control group without CNVs, and statistical significance was determined with a two-sided *Z* test. The significance level was set at 0.05.

## Results

### CNV analysis

**Fig. 1:**
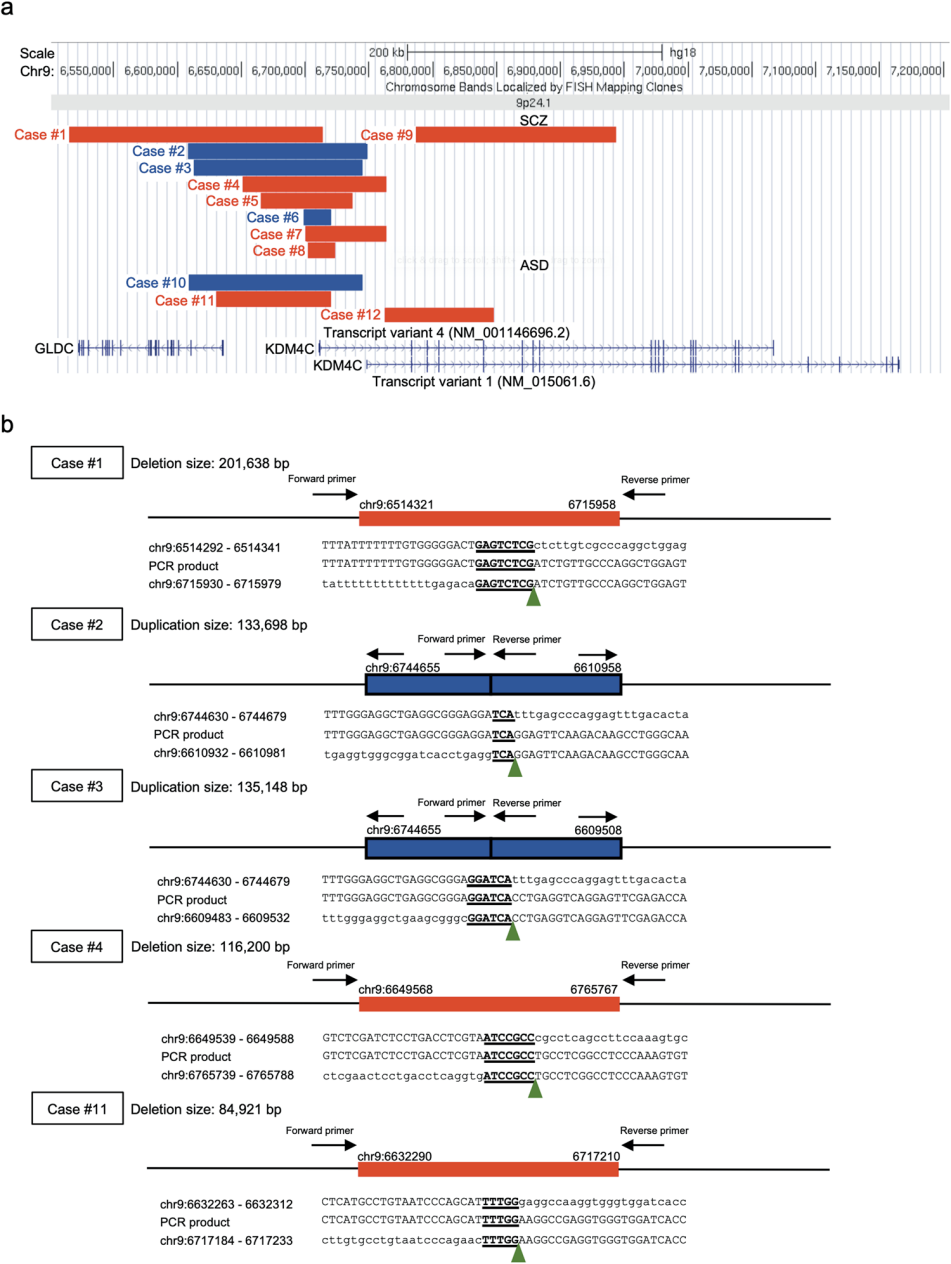
Exonic CNVs detected at *KDM4C* in patients with SCZ and ASD. **a** The location of exonic CNVs in *KDM4C*. The upper track shows the genomic position of the deletions (red bar) and duplications (blue bar). The lower track shows the gene annotations in RefSeq. The two transcript variants of *KDM4C* possessing different numbers of exons are depicted: transcript variant 4 (NM_ 001146696.2) and transcript variant 1 (NM_015061.6). Several other minor transcript variants of *KDM4C* were not shown (NM_001146695.4, NM_001304340.4, NM_001304339.4, NM_001304341.4, NM_001353997.3, NM_001353998.3, NM_001353999.3, NM_001354000.3, and NM_001354001.3). All the minor transcript variants have the same location of the first exon with the transcript variant 1. **b** Breakpoints of CNVs in *KDM4C* determined by Sanger sequencing. The red bars represent deletions and the blue bars represent duplications. Position of the breakpoints is marked by green arrowheads. The microhomologies were shown in underline. Genomic locations are given in NCBI build 36/UCSC hg18 coordinates. SCZ schizophrenia, ASD autism spectrum disorder.

We performed aCGH for 2810 SCZ cases, 1182 ASD cases, and 2428 controls. After QC, we obtained CNV data for 2605 SCZ cases, 1141 ASD cases, and 2310 controls. We identified exonic CNVs of *KDM4C* in nine SCZ cases and three ASD cases, but none in the controls (Fig. 1a and Table 1). We detected the novel CNVs in three SCZ patients by using moderate criteria for CNV calling and QC in an expanded sample set. Eight of 12 CNVs were deletions (six in SCZ and two in ASD) and the other four CNVs were duplications (three in SCZ and one in ASD). All the CNVs were validated using qPCR (Supplementary Fig. S1). The sizes of the CNVs were 22−198 kb. Two deletions (in cases #9 and #12) overlapped with the exons encoding the JmjC domain, which plays a central role in histone lysine demethylation. In case #9, the mRNA was predicted to result in the deletion of exons 3−8. In case #12, the deletion removed the 5′-untranslated region and the putative promoter region upstream of the transcription start site. The other two deletions (cases #4 and #7) overlapped with the first exon of *KDM4C* transcript variant 4 (RefSeq: NM_001146696.2, encoding an 835-amino acid variant) and transcript variant 1 (RefSeq: NM_015061.6, encoding a 1056-amino acid variant). All the other CNVs overlapped with only the first exon of transcript variant 4 (Fig. 1a). We found a significant association between *KDM4C* CNVs and SCZ (*p* = 0.003; OR = 16.9) and ASD (*p* = 0.04; OR = 14.2). Then we separately evaluated the association between such disorders and deletion and duplication. We observed significant association with deletions in the SCZ cases (corrected *p* = 0.04) but not for exonic duplications (corrected *p* = 0.30) after Bonferroni’s correction for multiple testing. Neither the frequency of exonic *KDM4C* deletions (corrected *p* = 0.22) nor that of *KDM4C* duplications (corrected *p* = 0.66) in the ASD cases differed significantly from that of the controls.

**Table 1:** Details of the detected CNVs in *KDM4C* and the clinical phenotype of each case.

Sample ID/Age (years old)/Sex	Diagnosis	CNVs	CNV region (size)	Exons affected	Protein region affected (aa)	Inheritance	Family history	Age at onset (years old)	Congenital and developmental phenotypes	Core symptoms	Epilepsy	Physical comorbidities	Brain imaging	Treatment	
														Antipsychotics (chlorpromazine equivalent)	Treatment resistance
Case #1/58/M	SCZ	Del	chr9:6515251−6713559 (198.3 kb)	1	1−17	NA	NA	NA	NA	NA	NA	NA	NA	NA	
Case #2/22/M	SCZ	Dup	chr9:6608534−6748433 (139.9 kb)	1	1−17	NA	–	10s	+ (mild intellectual disability)	Persecutory delusions, hallucination, disorganized speech, grossly disorganized behavior and cognitive decline	–	–	NA	1142 mg/day	+
Case #3/54/F	SCZ	Dup	chr9:6612895−6744567 (131.6 kb)	1	1−17	NA	NA	NA	NA	NA	NA	NA	NA	NA	
Case #4/42/M	SCZ	Del	chr9:6651146−6763692 (112.5 kb)	1	1−17	NA	SCZ (Brother)	30s	–	Persecutory delusions, hallucination, disorganized speech, grossly disorganized behavior and cognitive decline	–	Atrial septal defect (took operation during childhood)	NA	1300 mg/day	+
Case #5/31/M	SCZ	Del	chr9:6665064−6737056 (72.0 kb)	1	1−17	Paternal	–	20s	*–*	Persecutory delusions and hallucination	–	Hypertension	CT: mild frontal atrophy	750 mg/day	+
Case #6/29/F	SCZ	Dup	chr9:6699022−6720532 (21.5 kb)	1	1−17	NA	NA	NA	NA	NA	NA	NA	NA	NA	
Case #7/19/M	SCZ	Del	chr9:6700011−6763692 (63.7 kb)	1	1−17	Maternal	–	10s	–	Cognitive decline	–	–	MRI: no significant findings	150 mg/day	
Case #8/63/M	SCZ	Del	chr9:6701930−6723502 (21.6 kb)	1	1−17	NA	–	20s	–	Persecutory delusions, hallucination, disorganized speech, grossly disorganized behavior and catatonic behavior with mutism and stupor	–	–	CT: diffuse cortical atrophy	725 mg/day	+
Case #9/24/F	SCZ	Del	chr9:6786319−6943494 (157.2 kb)	3-8	71−329	NA	NA	NA	NA	NA	NA	NA	NA	NA	
Case #10/9/M	ASD	Dup	chr9:6608679−6744567 (135.9 kb)	1	1−17	NA	Broad autism phenotype (Mother)	<10	+ (ADHD)	Deficits in social communication and social interaction. Restricted, repetitive patterns of behavior, interests, or activities	–	–	NA	–	
Case #11/18/M	ASD	Del	chr9:6630498−6720532 (90.0 kb)	1	1−17	NA	ASD (Mother)	<10	–	Deficits in social communication and social interaction. Restricted, repetitive patterns of behavior, interests, or activities	–	–	NA	–	
Case #12/31/M	ASD	Del	chr9:6762250−6847825 (85.6 kb)	2−5	18−232	NA	–	<10	+ (severe intellectual disability)	Deficits in social communication and social interaction. Restricted, repetitive patterns of behavior, interests, or activities	+	–	CT: mild whole-brain atrophy	NA	

Genomic locations are given in NCBI build 36/UCSC hg18 coordinates. Exons and protein region affected by exonic CNVs are based on NM_001146696.1 and NP_001140168.1, respectively.

*aa* amino acid, *Del* deletion, *Dup* duplication, *SCZ* schizophrenia, *ASD* autism spectrum disorder, *ADHD* attention deficit hyperactivity disorder, *NA* not available.

Post-hoc calculations of statistical power showed that both of our SCZ and ASD samples had sufficient statistical power (1 − *β* > 80%) for CNVs with rare-allele frequencies of 0.34 and 0.26% detected within our SCZ and ASD samples respectively if the relative risk was >2.3 and >2.8 for each.

### Breakpoint analysis

The breakpoints of *KDM4C* CNVs were determined in five of 12 cases (three with a deletion and two with a duplication: Fig. 1b) using Sanger sequencing. Most of the detected breakpoints were different. Only the 3′ end breakpoints of two duplications (cases #2 and #3) were the same. Both duplications were confirmed to be tandem in direct orientation and adjacent to the original locus. We detected 3- to 8-bp microhomologies without insertions at all five CNV breakpoints. The results suggest that the possible mechanisms leading to the formation of the CNVs are nonhomologous end joining (NHEJ), microhomology-mediated end joining (MMEJ) or microhomology-mediated break-induced replication (MMBIR)^33^. No breakpoints involved fused genes in the same direction, and none were predicted to generate fusion transcripts.

### Evaluation of the clinical characteristics of patients with *KDM4C* CNVs

The clinical features of the cases with *KDM4C* exonic CNVs are shown in Table 1. Two cases (one SCZ and one ASD) had a family history of psychiatric disorders. Two patients had intellectual disability, and one patient had other psychiatric comorbidities. For physical comorbidities, epilepsy, an atrial septal defect, and hypertension were observed in one case. Brain atrophy was observed with brain imaging in three cases. About half of SCZ cases showed treatment resistance despite high-dose antipsychotics.

### Detection of SNVs and association analysis

**Fig. 2:**
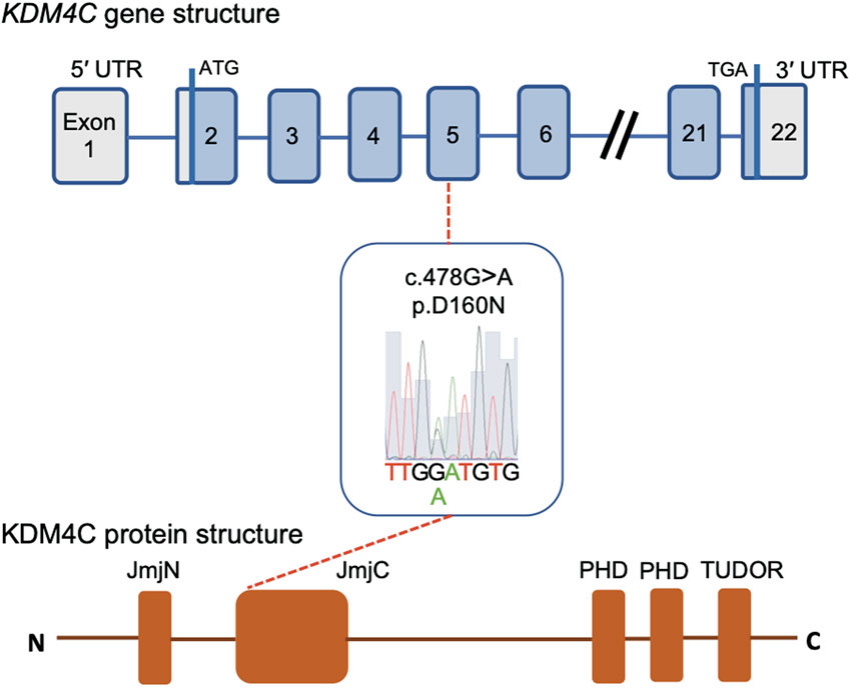
Location of novel rare variant. *KDM4C* gene structure is based on NM_015061.6; blue boxes indicate the protein-coding region. Gray boxes indicate the 5′ and 3′ untranslated regions (UTR). The localization of protein domain is based on the Pfam protein families database.

In the second sample set [SCZ or ASD patients (*n* = 562)], we identified 18 rare missense variants within *KDM4C* coding exons; five of them were predicted to be deleterious by SIFT or PolyPhen-2 (Table 2 and Supplementary Table S6). All SNVs were confirmed by Sanger sequencing, and all of them were heterozygous. Four of the five deleterious missense variants (p.T18S, p.D160N, p.R816Q, p.F907C) were located in a genomic region that is highly conserved among seven vertebrates (Supplementary Table S7). Among the four variants, one missense variant (p.D160N) was assumed to have the highest effect on SCZ because of the following findings: p.D160N is located in the JmjC domain, which plays a central role in histone lysine demethylation through an oxidative reaction (Fig. 2), and p.D160N was not registered in several public databases (Table 2). We therefore performed genetic association analysis for p.D160N. For the third sample set of 1751 SCZ cases and 2276 controls, we computed a statistical power of >80% using the following parameters: disease prevalence of 0.01, observed rare-allele frequency of 0.0027, OR for dominant effect of ≥2.6, and type I error rate of 0.05. We discovered p.D160N in three SCZ cases but in none of the healthy controls, however, we found no statistically significant association between p.D160N and SCZ (*p* = 0.08, Table 2). Nucleotide sequence data are available with the accession number DR004490DNA in the DNA Data Bank of Japan (DDBJ) databases (http://www.ddbj.nig.ac.jp).

**Table 2:** Details of the discovered rare missense variants in *KDM4C* predicted as pathogenic.

Physical position^a^	Transcript variant	Protein variant	Variant screening^b^ (second sample set)		Association analysis^b^ (third sample set)			Bioinformatical analysis		db SNP ID	Frequency of variants		
			SCZ: 370	ASD: 192	SCZ: 1751	ASD: 377	CON: 2276	SIFT	Polyphen-2		ToMMo 2k^c^	HGVD^c^	ExAC^c^
chr9:6793041	c.119C > G	p.T18S	1	0	–	–	–	Damaging	Possibly Damaging	–	4/4096	0	1/121,410
chr9:6849549	c.478G > A	p.D160N	1	0	3 (*p* = 0.08)	0 (*p* = 1.0)	0	Damaging	Possibly Damaging	–	0	0	0
chr9:6984183	c.1133C > T	p.S378F	1	0	–	–	–	Damaging	Benign	–	1/4096	0	2/120,958
chr9:7103707	c.2447G > A	p.R816Q	4	1	–	–	–	Tolerated	Probably Damaging	rs180710573	26/4094	12/875	8/121,228
chr9:7128175	c.2720T > G	p.F907C	3	1	–	–	–	Damaging	Probably Damaging	rs199621992	0	2/1192	9/121,196

*P*-values were calculated using Fisher’s exact test (2 × 2 contingency table, one-tailed).

*ToMMo* Tohoku Medical Megabank Organization, *HGVD* human genetic variation database, *ExAC* exome aggregation consortium

^a^Physical positions based on NCBI build 37/UCSC hg19.

^b^The number of samples.

^c^Minor allele count/total allele count.

### mRNA expression analysis using LCLs

**Fig. 3:**
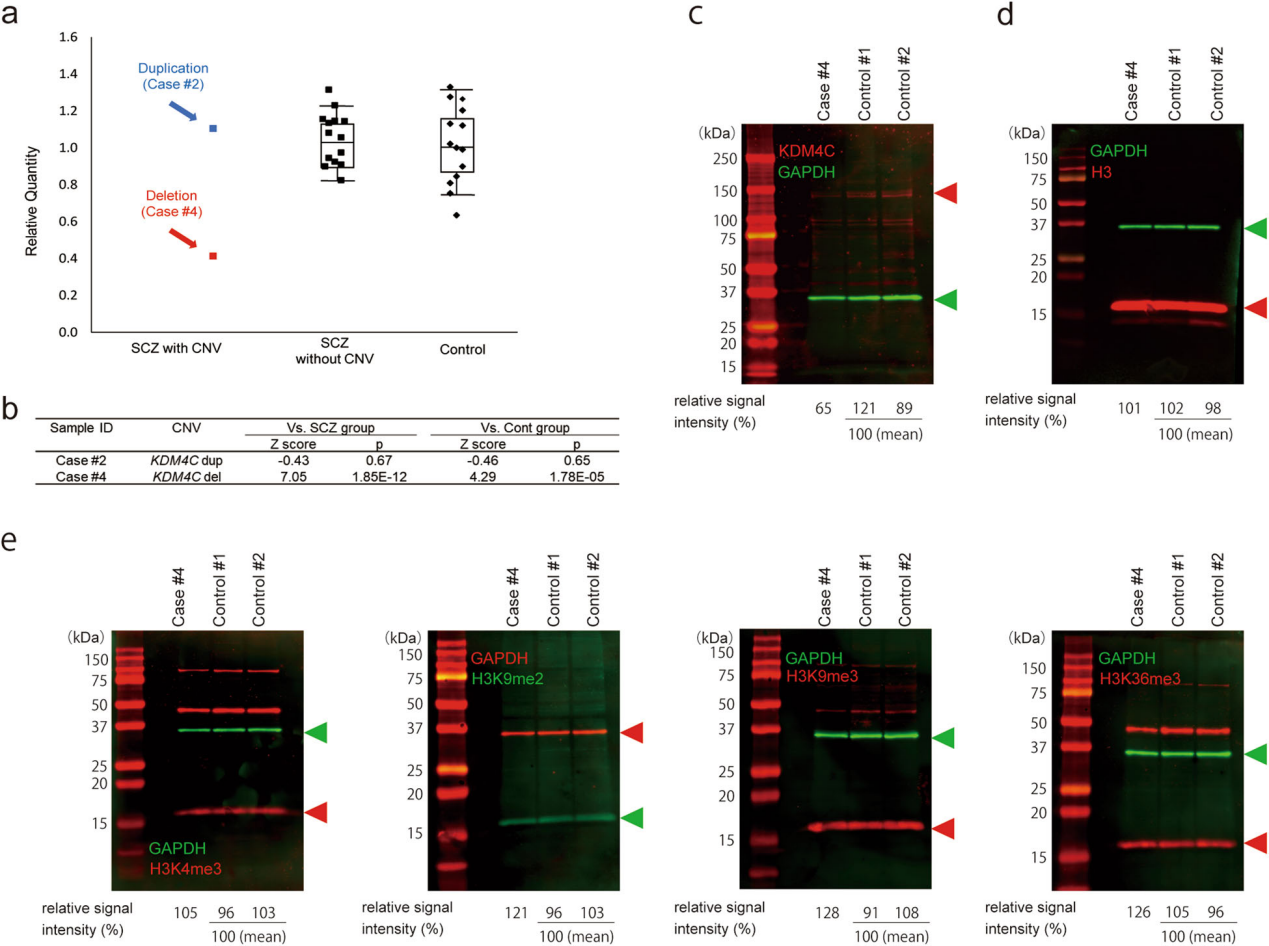
mRNA expression and immunoblotting analysis using LCLs. **a** *KDM4C* mRNA expression analysis. Boxplot; box represents the middle 50% of observations. The middle bold line represents the median gene expression. Whiskers represent the minimum and maximum observations (without outliers). Each dot represents the relative expression of an individual sample calculated by the 2^−ΔΔCt^ method. **b** Results of statistical tests for *KDM4C* mRNA expression. **c** Western blot analysis of KDM4C. **d** Western blot analysis of H3. **e** Immunoblotting analysis of histone lysine methylation status: H3K4me3, H3K9me2, H3K9me3, and H3K36me3.

To investigate the effects of *KDM4C* CNVs, we assessed the levels of *KDM4C* mRNA expression in LCLs derived from two patients with *KDM4C* CNVs (case #2 with a duplication and case #4 with a deletion). The mRNA transcript level was significantly lower in LCLs from the patient with the *KDM4C* deletion than in LCLs from SCZ patients without CNVs and healthy controls (*p* = 1.85 × 10^−12^, 1.78 × 10^−5^, respectively) (Fig. 3a, b).

### Immunoblotting analysis

We confirmed the association of SCZ with *KDM4C* deletion but not with *KDM4C* duplication. Furthermore, we revealed that *KDM4C* mRNA expression is significantly changed in LCLs derived from a case with *KDM4C* deletion but not in LCLs from a case with *KDM4C* duplication. Therefore, we analyzed KDM4C protein expression levels and histone lysine methylation patterns of H3K9, H3K36 and H3K4 by using the LCLs derived from the SCZ patient with *KDM4C* deletion (case #4). KDM4C protein expression was decreased by 35% in LCLs with *KDM4C* deletion (Fig. 3c). We confirmed elevation of the levels of H3K9me2 (21%), H3K9me3 (28%) and H3K36me3 (26%), all of which are demethylated by KDM4C, whereas the level of H3K4me3 in LCLs with *KDM4C* deletion was similar to that in LCLs derived from the controls. The relative signal intensity was 105% (Fig. 3e).

## Discussion

In the present study with an expanded sample (*N* = 6056), we identified rare CNVs of *KDM4C* in 12 patients with SCZ or ASD, but not in controls. We found a separate, significant genetic association between the *KDM4C* CNVs and SCZ and ASD. Thus, *KDM4C* CNVs increase the risk of the development of SCZ and ASD. This result is consistent with the recent study of CNVs in a large sample size of SCZ and ASD cases; some rare CNVs in specific loci are shared risk factors for both SCZ and ASD^3,34^. Ten of 12 CNVs overlapped with exon 1 of *KDM4C* transcript variant 4 (NM_001146696.2; Fig. 1a). Only four of 12 CNVs overlapped with the exons of *KDM4C* transcript variant 1 (NM_015061.6; Fig. 1a). We speculated that disruption of variant 4 increases susceptibility to the development of SCZ and ASD. However, the Genotype-Tissue Expression (GTEx) project^35^ (http://www.gtexportal.org) reported that variant 4 is mainly expressed in the liver and the testis, whereas variant 1 is expressed in the various organs, including the brain. Considering that variant 1 is expressed in the human brain from the fetus to the adult^25^, and that variant 4 lacks the tandem Tudor domain which is essential for KDM4C to recognize H3K4me3^36^, variant 1 may be important for brain development. In case #9, the deletion results in a protein variant that lacks the JmjC domain. In cases #4, #7, and #12, the deletion removes the transcription start site and the promoter of variant 1, which may affect transcription. The other CNVs overlapped with the putative enhancer region of variant 1 as indicated by the GeneHancer online database^37^; thus, these CNVs may affect the expression of variant1. However, which variant plays a pivotal role in neural development is still controversial. Further research is needed.

The CNV breakpoint analysis at the sequence level detected 3- to 8-bp microhomologies without insertions at these junctions (Fig. 1b). MHEJ is thought to require shorter stretches of microhomology (1−4 bp) than MMEJ (>5 bp)^33^. The possible mutational mechanisms leading to the formation of CNVs are NHEJ or MMBIR in case #2, and MMEJ or MMBIR in cases #1, #3, #4, and #11.

Evaluation of the clinical characteristics of patients with *KDM4C* CNVs revealed that about half of SCZ cases showed treatment resistance despite high-dose antipsychotics. *KDM4C* is located on 9p24.1. This locus is included in the potential ASD locus (9p24.3-9p23) in 9p duplication syndrome^38,39^. 9p duplication syndrome is clinically characterized by a variable degree of intellectual disabilities, craniofacial malformations such as microcephaly, down slanting palpebral fissures, hypertelorism, and distal phalangeal hypoplasia^40^. Several 9p duplication syndrome cases with SCZ or ASD have been reported^38,39^. Previously reported *KDM4C* CNVs in SCZ or ASD patients are summarized in Supplementary Fig. S2.

We also detected 18 rare missense variants. One missense variant, p.D160N, which was detected in a SCZ case, is located in the JmjC domain with a histone demethylase activity. Through association analysis, p.D160N was present exclusively in SCZ cases, but no significant association was detected between p.D160N and SCZ, perhaps because the size of our samples resulted in insufficient power.

Furthermore, we found decreased expression of *KDM4C* mRNA in LCLs established from an SCZ patient with *KDM4C* deletion. We found decreased KDM4C protein expression and elevation of the levels of H3K9me2, H3K9me3 and H3K36me3 in LCLs derived from this case with deletion. These three histone lysine methylation markers are demethylated by KDM4C. In contrast, a smaller change was observed in the level of H3K4me3, which recruits and stimulates KDM4C to demethylate H3K9^36^. These results suggest that decreased KDM4C expression changed the histone lysine methylation patterns of H3K9 and H3K36 in LCLs derived from an SCZ case with *KDM4C* deletion. Considering that KDM4C expression was decreased in the SCZ patient with *KDM4C* deletion and that *KDM4C* is expressed in the human brain from the fetus to the adult^25^ (Supplementary Fig. S3), KDM4C in SCZ patients with *KDM4C* deletion may have been decreased from the early fetal period. *KDM4C* deletion can potentially induce haploinsufficiency of KDM4C and affect brain development.

The CNVs also overlapped with glycine decarboxylase (*GLDC*) in five cases (one SCZ case and one ASD case with deletion, and two SCZ cases and one ASD case with duplication: Fig. 1a). *GLDC* encodes an enzyme that catabolizes glycine, which is a co-agonist at *N*-methyl-d-aspartate receptors. Reduced availability of glycine results in hypofunction of these receptors and is related to the pathophysiology of SCZ^41^. Recently, several CNVs including *GLDC* triplication and *KDM4C* partial triplication were detected in a proband and his mother who were diagnosed with schizoaffective disorder and bipolar disorder with psychotic features, respectively^42^. Glycine or d-cycloserine augmentation of psychotropic drug treatment each improved their psychotic and mood symptoms^42^. In our samples, one case had full deletion of *GLDC*, and the other *GLDC* CNVs were partial deletions or partial duplications. No statistically significant association between *GLDC* CNVs and SCZ and ASD was found in our previous study^3^. *GLDC* mRNA expression was not elevated in LCLs derived from the patient with partial duplication of *KDM4C* and *GLDC* (Supplementary Fig. S4).

*KDM4C* and *KDM4A* control intrinsic glial fibrillary acidic protein (*GFAP*) expression and astrocyte differentiation in neural progenitors and are selectively associated with the methylation patterns of H3K36 ^26^. GFAP-positive astrocytes are increased in the brain of *Kdm4c* hypomorphic mutant mice, which have phenotypic features that resemble the characteristics of developmental disorders including ASD^27^. This observation is interestingly consistent with GFAP elevation in the brain tissue of patients with ASD^43,44^. Several studies suggest that astrocytes may also contribute to the pathology of SCZ^45,46^. Recently, Chip-seq analysis using B cells revealed that KDM4C binds several genes related to neural development^47^. One of the genes is *AUTS2*, which plays a pivotal role during neuronal migration and has emerged as a crucial gene associated with SCZ and ASD^48^. Still, little is understood about the physiological functions of KDM4C in neural development and the mechanism by which the risk of SCZ and ASD is increased by *KDM4C* CNVs. Further research is needed to reveal how KDM4C regulates histone lysine methylation and spatio-temporal expression of other genes during neural development, and how these events contribute to neurogenesis.

Our study has several limitations. First, due to the difficulty of collecting samples from family members, we could not confirm whether the CNVs were de novo. Second, due to the insufficient power of our sample size, we could neither confirm nor dismiss the significance of p.D160N in SCZ and ASD. A third limitation is that we used LCLs derived from patients with SCZ, but not neural cells or glial cells, for immunoblotting analysis even though *KDM4C* is highly expressed both in brain and Epstein−Barr virus transformed lymphocytes^35^. Further analyses using neural cells and glial cells induced by stem cells or derived from animal models are needed.

In conclusion, we found significant genetic associations between *KDM4C* CNVs and SCZ and ASD. We also confirmed the significant association between *KDM4C* deletion and SCZ. Ours is the first report to describe the phenotypic features of SCZ and ASD patients with *KDM4C* CNVs. We found decreased gene expression and changes in histone methylation patterns in LCLs established from a SCZ patient with *KDM4C* deletion. *KDM4C* deletion may confer susceptibility to the pathogenesis of SCZ through haploinsufficiency of KDM4C. In future research, how KDM4C regulates the spatio-temporal expression of other genes during neural development and how KDM4C contributes to neurogenesis should be examined.

## Supplementary information

Supplement
