# Supplementary material for: Rare genetic variants in the gene encoding histone lysine demethylase 4C (KDM4C) and their contributions to susceptibility to schizophrenia and autism spectrum disorder
Source: Transl Psychiatry. 2020 Dec 5;10:421. doi: 10.1038/s41398-020-01107-7 (PMC7719193; doi:10.1038/s41398-020-01107-7)
Supplement: Supplementary file 1 — Supplement [file 41398_2020_1107_MOESM1_ESM.docx]

***Supplementary Information***

***Rare Genetic Variants in the Gene Encoding Histone Lysine Demethylase 4C (KDM4C) and Their Contributions to Susceptibility to Schizophrenia and Autism Spectrum Disorder***

Hidekazu Kato^1^, MD, Itaru Kushima*^1,2^, MD, PhD, Daisuke Mori^1,3^, PhD, Akira Yoshimi^4^, PhD, Branko Aleksic^1^, MD, PhD, Yoshihiro Nawa^1^, MD, Miho Toyama^1^, MMedSc, Sho Furuta^1^, MD, Yanjie Yu^1^, MD, PhD, Kanako Ishizuka^1^, MD, PhD, Hiroki Kimura^1^, MD, PhD, Yuko Arioka^1,5^, PhD, Keita Tsujimura^1,6^, PhD, Mako Morikawa^1^, MD, PhD, Takashi Okada^1^, MD, PhD, Toshiya Inada^1^, MD, PhD, Masahiro Nakatochi^7^, PhD, Keiko Shinjo^8^, MD, PhD, Yutaka Kondo^8^, MD, PhD, Kozo Kaibuchi^9^, MD, PhD, Yasuko Funabiki^10^, MD, PhD, Ryo Kimura^11^, MD, PhD, Toshimitsu Suzuki^12,13^, PhD, Kazuhiro Yamakawa^12,13^, PhD, Masashi Ikeda^14^, MD, PhD, Nakao Iwata^14^, MD, PhD, Tsutomu Takahashi^15, 16^, MD, PhD, Michio Suzuki^15, 16^, MD, PhD, Yuko Okahisa^17^, MD, PhD, Manabu Takaki^17^, MD, PhD, Jun Egawa^18^, MD, PhD, Toshiyuki Someya^18^, MD, PhD and Norio Ozaki^1^, MD, PhD

Affiliations:

1 Department of Psychiatry, Nagoya University Graduate School of Medicine, Nagoya, Japan

2 Medical Genomics Center, Nagoya University Hospital, Nagoya, Japan

3 Brain and Mind Research Center, Nagoya University, Nagoya, Japan

4 Division of Clinical Sciences and Neuropsychopharmacology, Faculty and Graduate School of Pharmacy, Meijo University, Nagoya, Japan

5 Center for Advanced Medicine and Clinical Research, Nagoya University Hospital, Nagoya, Japan

6 Innovative Research Unit for Developmental Disorders, Institute of Advanced Research, Nagoya University, Nagoya, Japan

7 Public Health Informatics Unit, Department of Integrated Health Sciences, Nagoya University Graduate School of Medicine, Nagoya, Japan

8 Division of Cancer Biology, Nagoya University Graduate School of Medicine, Nagoya, Japan

9 Department of Cell Pharmacology, Nagoya University Graduate School of Medicine, Nagoya, Japan

10 Department of Cognitive and Behavioral Science, Graduate School of Human and Environmental Studies, Kyoto University, Kyoto, Japan

11 Department of Anatomy and Developmental Biology, Graduate School of Medicine, Kyoto University, Kyoto, Japan

12 Department of Neurodevelopmental Disorder Genetics, Institute of Brain Science, Nagoya City University Graduate School of Medical Sciences, Nagoya, Japan

13 Laboratory for Neurogenetics, RIKEN Center for Brain Science, Saitama, Japan

14 Department of Psychiatry, Fujita Health University School of Medicine, Toyoake, Japan

15 Department of Neuropsychiatry, University of Toyama Graduate School of Medicine and Pharmaceutical Sciences, Toyama, Japan

16 Research Center for Idling Brain Science, University of Toyama, Toyama, Japan

17 Department of Neuropsychiatry, Okayama University Graduate School of Medicine, Dentistry and Pharmaceutical Sciences, Okayama, Japan

18 Department of Psychiatry, Niigata University Graduate School of Medical and Dental Sciences, Niigata, Japan

*Corresponding author:

Itaru Kushima, MD, PhD

Department of Psychiatry, Nagoya University Graduate School of Medicine

65 Tsurumai-cho, Showa-ku, Nagoya, Aichi-ken 466-8550, Japan

Tel: +81 52 7442282; Fax: +81 52 7442293

E-mail: [kushima@med.nagoya-u.ac.jp](mailto:kushima@med.nagoya-u.ac.jp)

**Contents of Supplementary Materials**

**Table S1. Primer sequences for validating CNVs**

**Table S2. Primer sequences for validating CNV breakpoints**

**Table S3. Primer sequences for validating each SNV**

**Table S4. Probe sequences for TaqMan SNP assays**

**Table S5. Primer sequences for mRNA expression analysis**

**Table S6. Details of the discovered rare missense variants in *KDM4C* predicted as benign**

**Table S7. The results of evolutionary conservation analysis**

**Figure S1. The results of validation for CNVs**

**Figure S2. Reported SCZ and ASD cases with 9p imbalances overlapping *KDM4C***

**Figure S3. Trajectories of gene expression in the brain based on Human Brain Transcriptome**

**Figure S4.** **The results of *GLDC* expression analysis in the patient with CNV**

**Table S1.** Primer sequences for validating CNVs

**
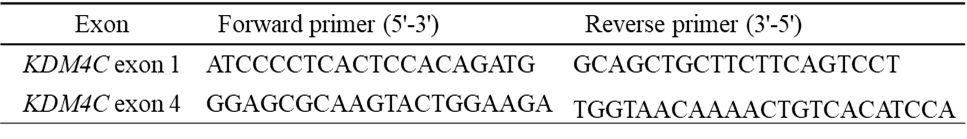
**

**Note:** The exon numbers are based on NCBI reference sequence: NM_001146696.2.

**Table S2.** Primer sequences for validating the CNV breakpoints


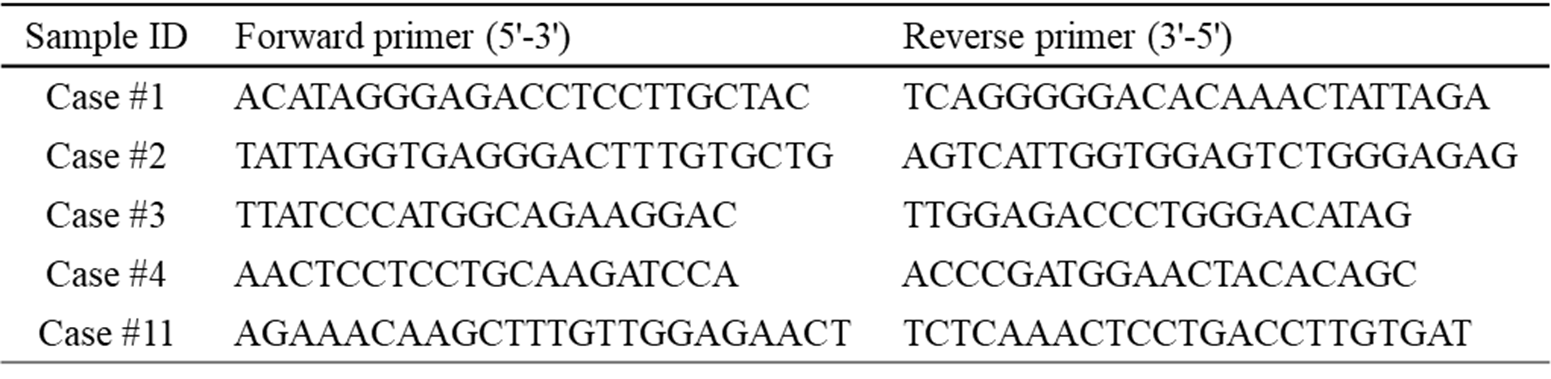


**Table S3.** Primer sequences for validating each SNV

**
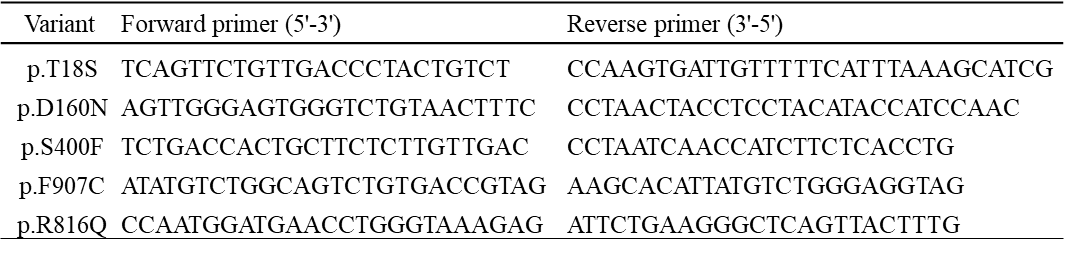
**

**Note:** Position of each variant is based on NCBI reference sequence: NP_001140168.1.

**Table S4.** Probe sequences for TaqMan SNP assays

**
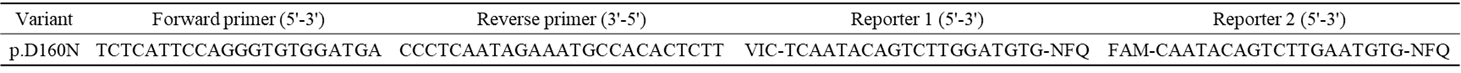
**

**Note:** A TaqMan probe consists of a VIC dye label on the 5' end and a nonfluorescent quencher (NFQ) on the 3' end.

**Table S5.** Primer sequences for mRNA expression analysis

**
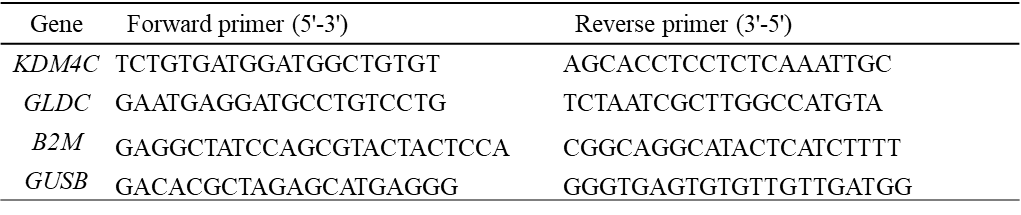
**

**Table S6.** Details of the discovered rare missense variants in *KDM4C* predicted as benign

**
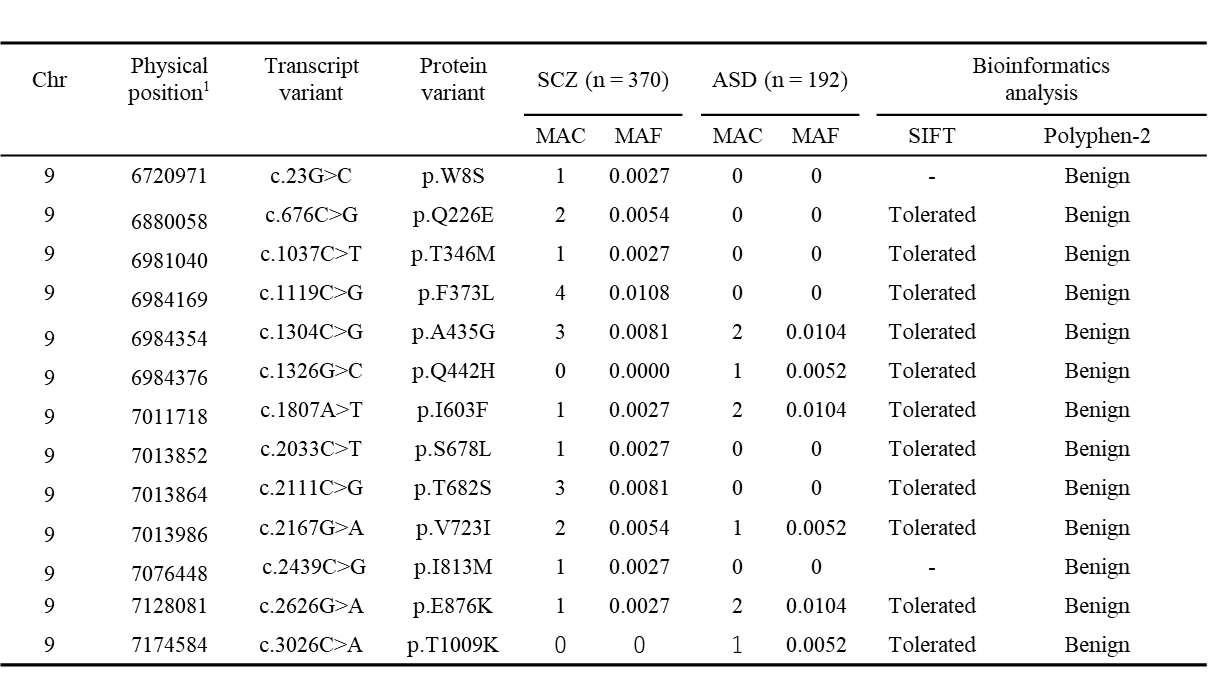
**

**Note:** Abbreviation: MAC, minor allele count; MAF minor allele frequency

^１^ Physical positions based on NCBI build GRCh37/hg19

**Table S7.** The results of evolutionary conservation analysis

**
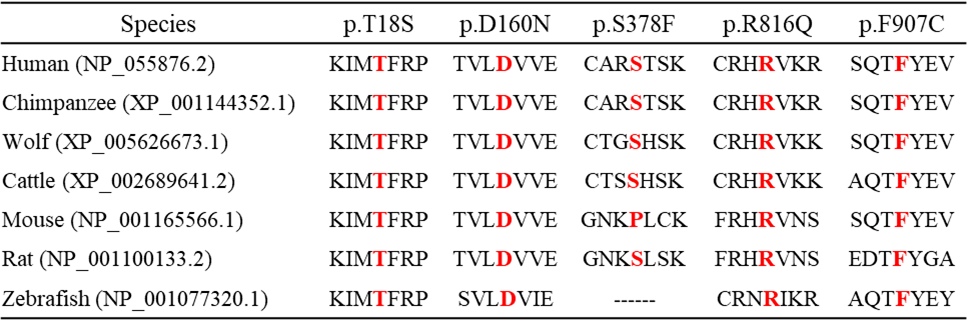
**

**Figure S1.** The results of validation for CNVs


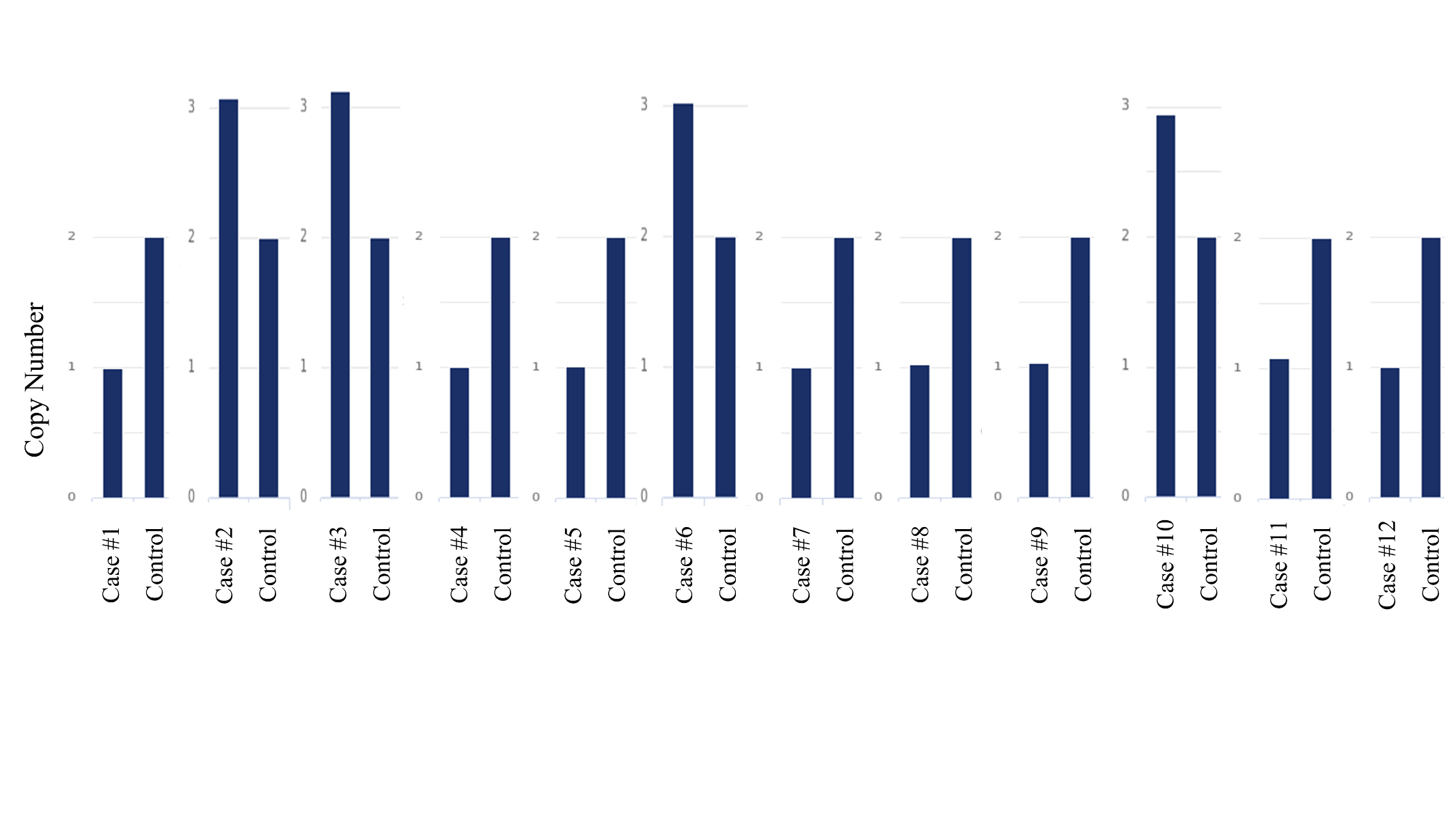


**Figure S2.** Reported SCZ and ASD cases with 9p imbalances overlapping *KDM4C*

*
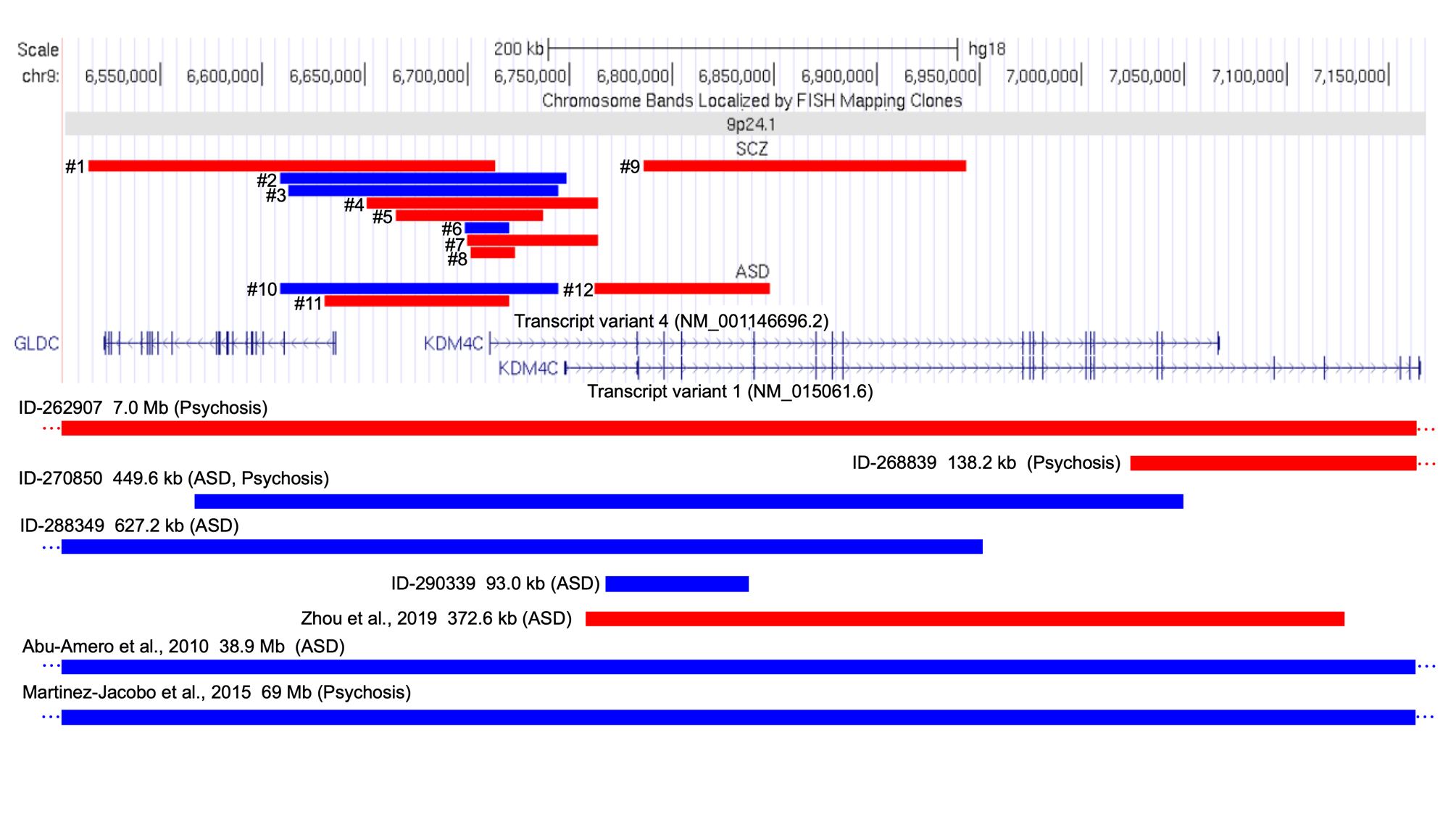
*

**Note:** The location of the CNVs in relation to the exons of *KDM4C* in the UCSC Genome Browser (http://genome.ucsc.edu) is illustrated. The red bars represent deletions and the blue bars represent duplications. The upper track shows the genomic position of the CNVs detected in the present study. The middle track shows the gene annotations in RefSeq. The lower track shows the reported cases with CNVs overlapping *KDM4C*. ID-262907, ID-268839, ID-270850, ID-288349, and ID-290339 are cases found in the DECIPHER database. Abbreviations: SCZ, schizophrenia; ASD, autism spectrum disorder

**Figure S3.** Trajectories of gene expression in the brain based on Human Brain Transcriptome

**
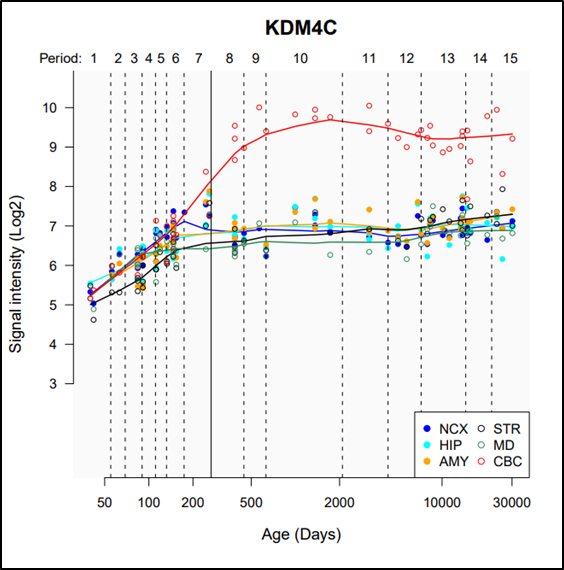
**

**Note:** This figure shows trajectories of exon array-based measurement of gene expression (mRNA level) in the brain by the Human Brain Transcriptome. *KDM4C* shows a gradual increase from early embryonic periods and then reaches a plateau. *KDM4C* expression is higher in the CBC than in other areas from the late fetus to the adult.

Abbreviations: NCX, neocortex; STR, striatum; HIP, hippocampus; MD, mediodorsal nucleus of the thalamus; AMY, amygdala; CBC, cerebellar cortex

**Figure S4. The results of *GLDC* expression analysis in the patient with CNV**

**a**

**
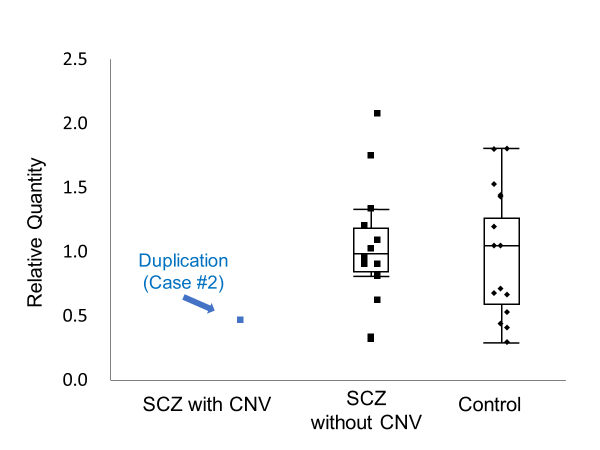
**

**b**

**Note:** (a) *GLDC* mRNA expression analysis. Each symbol [Blue square: sample with duplication, black squares: schizophrenia (SCZ), and diamonds: controls] represents the relative expression value of each sample calculated with the 2^−ΔΔCt^ method. Boxplot; Boxes represents the middle 50% of observations. The middle bold line represents the median gene expression. Whiskers represent the minimum and maximum observations (without outliers). (b) Results of statistical tests. Abbreviations: SCZ, schizophrenia; CNV, copy number variant; dup, duplication
